# Supplementary material for: A Likelihood Approach for Real-Time Calibration of Stochastic Compartmental Epidemic Models
Source: PLoS Comput Biol. 2017 Jan 17;13(1):e1005257. doi: 10.1371/journal.pcbi.1005257 (PMC5240920; doi:10.1371/journal.pcbi.1005257)
Supplement: S1 File — (TAR.GZ) [file pcbi.1005257.s014.tar.gz › HSPH_Online-SI-Revision/Additions/IPoi10/n100-extreme_noisy/n100-extreme_noisy_BENCH_table4.pdf]

1	R0	{2.76052, 16.3913}	{2.86118, 15.5722}	{{2.86118, 15.5722}, {2.86118, 15.5722}}	{3.64641, 4.99716}	inf	11 577.	12 599.2	{6201., 21 165.}	37.5849	0.154083	newly	4368.49	4938.65	{2372., 8400.}	40.3846	0.158929	Reff	0.335762	0.344899	{0.249695, 0.4862}	22.7665	ar	92 090.	93 013.	{92 837., 93 174.}	1.00228
2	R0	{2.18202, 15.1734}	{2.86095, 19.9929}	{{2.86095, 19.9929}, {2.86095, 19.9929}}	{31.1147, 31.7628}	inf	9523.	9080.97	{4391., 14 955.}	39.5516	0.180169	newly	3796.	2877.34	{1359., 5298.}	43.5594	0.239574	Reff	0.491915	0.338965	{0.235656, 0.392036}	50.7778	ar	84 264.	92 986.7	{92 779., 93 173.}	10.3517
3	R0	{2.57891, 12.5971}	{2.95376, 15.278}	{{2.95376, 15.278}, {2.95376, 15.278}}	{14.5352, 21.2819}	inf	6810.	10 792.2	{5468., 22 011.}	68.1855	0.192875	newly	3181.	4268.33	{2096., 8903.}	54.6241	0.174797	Reff	0.320791	0.297893	{0.226967, 0.478214}	26.7986	ar	90 302.	93 737.6	{93 580., 93 860.}	3.80458
4	R0	{2.5856, 9.28508}	{2.83858, 10.6973}	{{2.83858, 10.6973}, {2.83858, 10.6973}}	{9.78419, 15.2096}	inf	4198.	8270.53	{4677., 14 447.}	97.9496	0.257369	newly	2428.	4331.65	{2406., 7748.}	80.4834	0.217124	Reff	0.287622	0.282703	{0.240257, 0.360698}	13.9274	ar	90 312.	92 843.9	{92 700., 92 981.}	2.8035
5	R0	{2.4406, 12.1341}	{2.95376, 15.278}	{{2.95376, 15.278}, {2.95376, 15.278}}	{21.026, 25.9096}	inf	8911.	12 178.3	{6714., 19 069.}	47.9336	0.160075	newly	4440.	4783.79	{2553., 7533.}	35.8054	0.148621	Reff	0.386079	0.307296	{0.234942, 0.39985}	25.2826	ar	88 597.	93 731.7	{93 517., 93 893.}	5.79558
6	R0	{2.72155, 15.4094}	{2.8939, 16.8013}	{{2.8939, 16.8013}, {2.8939, 16.8013}}	{6.33279, 9.0328}	inf	11 194.	9597.05	{5947., 14 782.}	33.9409	0.165408	newly	4357.	3477.16	{2094., 5312.}	36.8504	0.189525	Reff	0.34629	0.29041	{0.243985, 0.350914}	23.4766	ar	91 682.	93 246.3	{93 116., 93 378.}	1.70618
7	R0	{2.63781, 14.3935}	{2.96978, 18.8857}	{{2.96978, 18.8857}, {2.96978, 18.8857}}	{12.5851, 31.2099}	inf	9306.	16 031.5	{9492., 24 792.}	73.2791	0.214486	newly	3953.	5363.27	{3114., 8396.}	45.4408	0.150973	Reff	0.327906	0.409362	{0.263865, 0.561823}	37.5597	ar	91 192.	93 838.8	{93 690., 93 998.}	2.9024
8	R0	{2.82391, 16.104}	{2.8939, 16.8013}	{{2.8939, 16.8013}, {2.8939, 16.8013}}	{2.47848, 4.32998}	inf	11 648.	12 495.	{7342., 20 033.}	32.2981	0.131693	newly	4404.	4589.41	{2593., 7829.}	33.6976	0.140759	Reff	0.322067	0.352486	{0.2531, 0.47049}	26.4319	ar	92 664.	93 276.	{93 124., 93 462.}	0.660498
9	R0	{2.80645, 14.6457}	{2.8939, 16.8013}	{{2.8939, 16.8013}, {2.8939, 16.8013}}	{3.11604, 14.7183}	inf	11 375.	16 697.	{9474., 26 925.}	52.9138	0.170231	newly	4649.	6209.38	{3427., 10 469.}	44.7652	0.151532	Reff	0.325885	0.422965	{0.278914, 0.720292}	36.0065	ar	92 567.	93 247.1	{93 100., 93 397.}	0.734701
10	R0	{1.86213, 5.4271}	{2.80387, 10.6536}	{{2.80387, 10.6536}, {2.80387, 10.6536}}	{50.5733, 96.3037}	inf	1169.	6354.26	{3037., 10 764.}	443.563	0.669126	newly	1101.	3339.96	{1564., 5655.}	203.357	0.41153	Reff	0.480541	0.270707	{0.233114, 0.316669}	44.5872	ar	75 362.	92 528.6	{92 355., 92 707.}	22.7789
11	R0	{2.27349, 14.6338}	{2.99088, 18.9406}	{{2.99088, 18.9406}, {2.99088, 18.9406}}	{31.5546, 29.4305}	inf	9305.	12 169.4	{7043., 19 348.}	41.7226	0.141214	newly	3974.	3976.43	{2322., 6323.}	31.4309	0.135959	Reff	0.44615	0.299728	{0.236668, 0.4035}	35.4651	ar	85 805.	93 991.3	{93 864., 94 121.}	9.54053
12	R0	{2.77261, 14.1894}	{2.85582, 17.2484}	{{2.85582, 17.2484}, {2.85582, 17.2484}}	{3.00114, 21.5583}	inf	10 089.	16 517.	{9572., 26 892.}	66.8748	0.20121	newly	4154.	6054.68	{3370., 10 268.}	54.1636	0.171893	Reff	0.306207	0.454804	{0.282812, 0.77804}	51.097	ar	92 508.	92 997.2	{92 856., 93 166.}	0.528869
13	R0	{2.65402, 19.3935}	{2.93978, 19.9817}	{{2.93978, 19.9817}, {2.93978, 19.9817}}	{10.7671, 3.03297}	inf	15 461.	11 749.8	{6813., 19 503.}	34.9725	0.195937	newly	4998.	3668.44	{2102., 6210.}	36.9835	0.209029	Reff	0.43088	0.350319	{0.245413, 0.423475}	40.6917	ar	91 184.	93 630.5	{93 477., 93 742.}	2.68309
14	R0	{1.75297, 4.45354}	{2.7943, 13.5962}	{{2.7943, 13.5962}, {2.7943, 13.5962}}	{59.4038, 205.29}	inf	1073.	13 588.	{7285., 22 235.}	1166.36	1.06368	newly	1153.	6077.96	{3169., 10 172.}	427.143	0.676355	Reff	0.522297	0.397435	{0.279095, 0.547264}	36.3265	ar	71 263.	92 478.9	{92 321., 92 654.}	29.7712
15	R0	{1.99928, 7.1501}	{2.7943, 13.5962}	{{2.7943, 13.5962}, {2.7943, 13.5962}}	{39.7653, 90.154}	inf	2594.	11 228.	{6199., 17 560.}	332.846	0.600328	newly	1932.	4975.38	{2659., 7915.}	157.525	0.373236	Reff	0.446419	0.406603	{0.266381, 0.472125}	42.3058	ar	79 602.	92 447.4	{92 291., 92 600.}	16.137
16	R0	{2.67398, 10.3134}	{2.7943, 13.5962}	{{2.7943, 13.5962}, {2.7943, 13.5962}}	{4.49966, 31.8304}	inf	4744.	9092.54	{4792., 14 444.}	93.5036	0.239628	newly	2584.	4032.56	{2029., 6332.}	66.2864	0.183163	Reff	0.275794	0.332918	{0.252241, 0.368261}	26.3521	ar	91 289.	92 440.6	{92 237., 92 698.}	1.26147
17	R0	{2.44634, 16.3226}	{2.93978, 19.9817}	{{2.93978, 19.9817}, {2.93978, 19.9817}}	{20.1705, 22.4174}	inf	11 754.	19 770.7	{12 066., 28 434.}	70.3547	0.215491	newly	4470.	6465.15	{3737., 9794.}	51.9332	0.171601	Reff	0.431632	0.562475	{0.292479, 0.910538}	51.2311	ar	88 710.	93 633.1	{93 480., 93 782.}	5.54961
18	R0	{2.02168, 10.1676}	{2.97157, 19.3547}	{{2.97157, 19.3547}, {2.97157, 19.3547}}	{46.9852, 90.3566}	inf	6854.	19 357.	{12 237., 27 308.}	182.419	0.430775	newly	4020.	6440.39	{3940., 9609.}	63.1914	0.194168	Reff	0.515609	0.51205	{0.289401, 0.842648}	41.1979	ar	80 299.	93 857.4	{93 725., 93 992.}	16.8849
19	R0	{2.89969, 15.009}	{2.8939, 16.8013}	{{2.8939, 16.8013}, {2.8939, 16.8013}}	{0.199677, 11.9415}	inf	11 247.	17 769.3	{10 303., 26 301.}	61.6756	0.192831	newly	4512.	6658.09	{3689., 10 065.}	55.0036	0.17758	Reff	0.292405	0.47453	{0.289448, 0.726456}	62.9464	ar	93 349.	93 258.5	{93 104., 93 440.}	0.153734
20	R0	{2.68933, 7.34928}	{2.83858, 10.6973}	{{2.83858, 10.6973}, {2.83858, 10.6973}}	{5.54971, 45.5558}	inf	2277.	8767.64	{4825., 13 434.}	285.052	0.550139	newly	1542.	4601.21	{2463., 7094.}	198.392	0.437379	Reff	0.246316	0.2886	{0.246786, 0.33552}	17.8016	ar	91 574.	92 822.	{92 624., 93 013.}	1.36288
21	R0	{2.15352, 9.28074}	{2.7943, 13.5962}	{{2.7943, 13.5962}, {2.7943, 13.5962}}	{29.755, 46.4991}	inf	5169.	13 210.5	{6460., 19 406.}	156.309	0.3759	newly	3172.	5825.67	{2766., 8679.}	89.2067	0.253469	Reff	0.421315	0.369046	{0.265319, 0.467123}	22.1765	ar	83 845.	92 430.8	{92 255., 92 624.}	10.2401
22	R0	{2.21953, 9.04387}	{2.99939, 13.7076}	{{2.99939, 13.7076}, {2.99939, 13.7076}}	{35.1363, 51.5679}	inf	4637.	11 805.7	{7051., 18 759.}	154.597	0.366842	newly	2827.	5196.72	{2991., 8266.}	86.4144	0.228064	Reff	0.397451	0.335318	{0.232303, 0.376603}	42.1955	ar	84 856.	94 032.2	{93 887., 94 187.}	10.8138
23	R0	{2.21987, 14.5347}	{2.86095, 19.9929}	{{2.86095, 19.9929}, {2.86095, 19.9929}}	{28.8792, 37.5529}	inf	9287.	14 075.1	{8212., 22 292.}	57.1664	0.17764	newly	3912.	4484.31	{2517., 7151.}	36.4779	0.139037	Reff	0.465951	0.406813	{0.274222, 0.585207}	38.1024	ar	85 067.	93 008.	{92 833., 93 186.}	9.33505
24	R0	{2.84268, 17.9659}	{2.76795, 19.6041}	{{2.76795, 19.6041}, {2.76795, 19.6041}}	{2.62886, 9.11839}	inf	14 057.	14 190.	{9231., 21 865.}	30.2612	0.141935	newly	4888.	4605.66	{2973., 7137.}	31.4447	0.152897	Reff	0.351242	0.456954	{0.3068, 0.661734}	39.1675	ar	92 836.	92 213.4	{92 040., 92 429.}	0.670674
25	R0	{2.52706, 10.3679}	{2.8833, 17.1847}	{{2.8833, 17.1847}, {2.8833, 17.1847}}	{14.097, 65.7491}	inf	5201.	15 140.	{8991., 21 161.}	191.098	0.437195	newly	2743.	5496.86	{3168., 7729.}	101.367	0.275178	Reff	0.316085	0.399967	{0.272904, 0.48921}	33.8635	ar	89 623.	93 156.2	{93 036., 93 276.}	3.94226
26	R0	{2.62835, 19.1088}	{2.93978, 19.9817}	{{2.93978, 19.9817}, {2.93978, 19.9817}}	{11.8489, 4.56805}	inf	14 411.	13 683.1	{8454., 18 626.}	24.3061	0.113021	newly	4701.	4283.31	{2602., 5864.}	25.3762	0.122338	Reff	0.418354	0.330058	{0.25432, 0.402573}	24.804	ar	90 908.	93 625.4	{93 459., 93 756.}	2.98913
27	R0	{2.76831, 19.4155}	{2.85582, 17.2484}	{{2.85582, 17.2484}, {2.85582, 17.2484}}	{3.16113, 11.1617}	inf	12 467.	6964.69	{3938., 11 696.}	48.3476	0.310432	newly	3937.	2448.81	{1349., 4178.}	45.3951	0.277181	Reff	0.347063	0.263347	{0.230807, 0.313341}	25.9599	ar	92 175.	92 997.8	{92 841., 93 140.}	0.892628
28	R0	{2.96609, 16.8903}	{2.7943, 13.5962}	{{2.7943, 13.5962}, {2.7943, 13.5962}}	{5.7918, 19.5029}	inf	11 510.	9104.6	{4653., 15 491.}	38.4996	0.207514	newly	4186.	3952.21	{1982., 6874.}	38.4465	0.17846	Reff	0.274037	0.30668	{0.247743, 0.380332}	16.6494	ar	94 024.	92 433.6	{92 235., 92 581.}	1.69151
29	R0	{2.17046, 14.39}	{2.76795, 19.6041}	{{2.76795, 19.6041}, {2.76795, 19.6041}}	{27.5283, 36.2342}	inf	10 054.	15 921.6	{10 122., 24 484.}	60.4045	0.187254	newly	4398.	5191.73	{3216., 8249.}	32.9553	0.122418	Reff	0.504914	0.470146	{0.3161, 0.720663}	34.2052	ar	84 001.	92 187.4	{92 067., 92 338.}	9.74555
30	R0	{2.88772, 12.4647}	{2.83858, 10.6973}	{{2.83858, 10.6973}, {2.83858, 10.6973}}	{1.70169, 14.1792}	inf	8175.	5938.37	{2526., 9301.}	47.262	0.270308	newly	3775.	3145.81	{1291., 4802.}	48.3305	0.246359	Reff	0.263591	0.267441	{0.221069, 0.287974}	16.8121	ar	93 273.	92 836.6	{92 671., 92 996.}	0.467925
31	R0	{2.46831, 8.14308}	{2.9194, 10.1535}	{{2.9194, 10.1535}, {2.9194, 10.1535}}	{18.2753, 24.6887}	inf	3768.	9569.64	{4777., 18 456.}	154.418	0.351061	newly	2494.	5314.64	{2532., 10 244.}	115.939	0.277732	Reff	0.317474	0.302039	{0.229494, 0.39914}	28.167	ar	88 762.	93 472.6	{93 333., 93 611.}	5.30696
32	R0	{1.93236, 7.38987}	{2.99939, 13.7076}	{{2.99939, 13.7076}, {2.99939, 13.7076}}	{55.219, 85.4918}	inf	3204.	11 675.2	{6956., 17 320.}	264.395	0.535477	newly	2353.	5003.55	{2915., 7460.}	112.66	0.299061	Reff	0.482588	0.28923	{0.232603, 0.356118}	40.1069	ar	77 654.	94 025.8	{93 886., 94 180.}	21.083
33	R0	{2.82251, 17.6954}	{2.86118, 15.5722}	{{2.86118, 15.5722}, {2.86118, 15.5722}}	{1.37006, 11.9986}	inf	15 003.	12 693.1	{7239., 19 331.}	34.5976	0.177504	newly	5209.	4969.38	{2787., 7529.}	36.3284	0.167125	Reff	0.365938	0.346348	{0.262742, 0.439792}	24.1138	ar	92 745.	93 036.4	{92 897., 93 181.}	0.314188
34	R0	{1.87083, 12.3994}	{2.76795, 19.6041}	{{2.76795, 19.6041}, {2.76795, 19.6041}}	{47.953, 58.1052}	inf	8229.	14 049.1	{8652., 23 318.}	72.1844	0.212939	newly	4286.	4538.52	{2774., 7787.}	31.2227	0.127331	Reff	0.630488	0.404156	{0.296614, 0.613516}	40.6152	ar	76 282.	92 192.6	{92 034., 92 369.}	20.8576
35	R0	{2.95779, 16.3003}	{2.86118, 15.5722}	{{2.86118, 15.5722}, {2.86118, 15.5722}}	{3.26629, 4.46679}	inf	14 081.	11 900.7	{6518., 17 745.}	32.8547	0.169159	newly	5272.	4647.78	{2438., 7036.}	34.18	0.16933	Reff	0.314472	0.336351	{0.251555, 0.416388}	21.2953	ar	93 873.	93 021.5	{92 864., 93 183.}	0.907036
36	R0	{2.18718, 14.261}	{2.93371, 17.1903}	{{2.93371, 17.1903}, {2.93371, 17.1903}}	{34.1321, 20.5406}	inf	9571.	6272.43	{3339., 10 166.}	46.0711	0.272712	newly	4189.	2207.74	{1231., 3554.}	53.9626	0.354496	Reff	0.488835	0.243543	{0.21243, 0.277206}	50.4454	ar	84 379.	93 558.6	{93 410., 93 725.}	10.879
37	R0	{2.44912, 9.31118}	{2.9884, 12.8326}	{{2.9884, 12.8326}, {2.9884, 12.8326}}	{22.0193, 37.8193}	inf	4364.	13 134.5	{6680., 23 283.}	200.975	0.432337	newly	2540.	6131.44	{3021., 10 948.}	142.072	0.333498	Reff	0.332493	0.372196	{0.230495, 0.556978}	41.2561	ar	88 442.	93 969.3	{93 809., 94 125.}	6.24959
38	R0	{2.05949, 5.72514}	{2.9884, 12.8326}	{{2.9884, 12.8326}, {2.9884, 12.8326}}	{45.1039, 124.145}	inf	1447.	11 260.	{6605., 16 647.}	678.161	0.858251	newly	1229.	5132.02	{2993., 7669.}	317.577	0.588902	Reff	0.397749	0.32987	{0.230226, 0.352392}	39.5358	ar	81 587.	93 969.	{93 829., 94 166.}	15.1764
39	R0	{1.75505, 8.52404}	{2.97157, 19.3547}	{{2.97157, 19.3547}, {2.97157, 19.3547}}	{69.3154, 127.06}	inf	4589.	17 766.	{9969., 27 707.}	288.873	0.563607	newly	3152.	5833.7	{3155., 9606.}	88.958	0.253327	Reff	0.605615	0.475184	{0.266401, 0.741585}	45.3182	ar	70 925.	93 854.9	{93 720., 94 001.}	32.3298
40	R0	{2.24724, 13.7861}	{2.8939, 16.8013}	{{2.8939, 16.8013}, {2.8939, 16.8013}}	{28.7757, 21.8713}	inf	8230.	11 112.7	{5861., 19 476.}	53.4914	0.170727	newly	3630.	4087.02	{2142., 7228.}	47.2566	0.180293	Reff	0.451088	0.32863	{0.243145, 0.444532}	38.9674	ar	85 257.	93 278.	{93 105., 93 430.}	9.40806
41	R0	{1.77635, 8.62406}	{2.86118, 15.5722}	{{2.86118, 15.5722}, {2.86118, 15.5722}}	{61.0707, 80.5669}	inf	4190.	12 473.1	{6770., 21 527.}	197.688	0.432557	newly	2830.	4926.83	{2606., 9846.}	78.3669	0.213897	Reff	0.557952	0.368137	{0.255446, 0.602164}	45.487	ar	72 941.	93 037.3	{92 868., 93 204.}	27.5514
42	R0	{1.80397, 5.4932}	{2.91946, 11.8546}	{{2.91946, 11.8546}, {2.91946, 11.8546}}	{61.8353, 115.805}	inf	1509.	5283.72	{2829., 7661.}	250.147	0.506292	newly	1451.	2510.79	{1357., 3628.}	76.5669	0.215233	Reff	0.511444	0.233284	{0.211311, 0.253935}	54.3871	ar	73 232.	93 471.5	{93 365., 93 594.}	27.6375
43	R0	{2.83096, 16.7077}	{2.86489, 19.9923}	{{2.86095, 19.9929}, {2.86095, 19.9929}}	{1.19859, 19.6594}	inf	13 176.	19 205.6	{13 213., 27 297.}	47.6914	0.158431	newly	4750.	6194.67	{4117., 9423.}	36.8013	0.128235	Reff	0.342093	0.509085	{0.334245, 0.884549}	50.4376	ar	92 740.	93 043.2	{92 886., 93 204.}	0.330641
44	R0	{2.73678, 11.6021}	{2.9884, 12.8326}	{{2.9884, 12.8326}, {2.9884, 12.8326}}	{9.19402, 10.6058}	inf	5400.	5663.45	{3017., 10 020.}	39.8244	0.164993	newly	2650.	2526.18	{1325., 4437.}	39.2384	0.17871	Reff	0.263771	0.2251	{0.200581, 0.264653}	16.9736	ar	92 003.	93 955.7	{93 826., 94 096.}	2.12243
45	R0	{2.14235, 6.98601}	{2.7943, 13.5962}	{{2.7943, 13.5962}, {2.7943, 13.5962}}	{30.4315, 94.6204}	inf	1905.	12 503.8	{6984., 20 818.}	556.368	0.77848	newly	1420.	5563.52	{3032., 9414.}	291.797	0.547778	Reff	0.389458	0.376568	{0.271634, 0.501381}	28.6838	ar	82 974.	92 435.6	{92 223., 92 668.}	11.4031
46	R0	{2.23409, 9.21289}	{2.95376, 15.278}	{{2.95376, 15.278}, {2.95376, 15.278}}	{32.2131, 65.8329}	inf	5803.	16 658.9	{10 255., 24 090.}	187.073	0.437273	newly	3539.	6806.3	{4124., 9748.}	92.417	0.259774	Reff	0.407766	0.476706	{0.275497, 0.691298}	44.3687	ar	85 163.	93 724.2	{93 559., 93 924.}	10.0527
47	R0	{2.12258, 8.15265}	{2.9884, 12.8326}	{{2.9884, 12.8326}, {2.9884, 12.8326}}	{40.7909, 57.404}	inf	3887.	11 734.9	{6925., 19 114.}	201.901	0.442416	newly	2681.	5373.39	{3058., 8824.}	101.096	0.26291	Reff	0.420441	0.302497	{0.230256, 0.392138}	36.1007	ar	82 690.	93 972.	{93 798., 94 101.}	13.6437
48	R0	{2.71771, 10.4636}	{2.9884, 12.8326}	{{2.9884, 12.8326}, {2.9884, 12.8326}}	{9.96022, 22.6404}	inf	6665.	12 540.1	{7483., 21 170.}	89.5357	0.249106	newly	3585.	5815.86	{3333., 9930.}	66.5801	0.192226	Reff	0.28718	0.347757	{0.236861, 0.45086}	34.0827	ar	91 716.	93 971.9	{93 839., 94 093.}	2.45967
49	R0	{2.44369, 6.66477}	{2.98388, 9.60029}	{{2.98388, 9.60029}, {2.98388, 9.60029}}	{22.1055, 44.0453}	inf	1262.	5146.92	{2842., 9003.}	307.838	0.56653	newly	921.	2862.33	{1559., 5072.}	210.785	0.446459	Reff	0.285399	0.221399	{0.198189, 0.254018}	22.6472	ar	88 827.	93 913.	{93 768., 94 067.}	5.72577
50	R0	{2.77383, 17.9775}	{2.97157, 19.3547}	{{2.97157, 19.3547}, {2.97157, 19.3547}}	{7.12877, 7.66069}	inf	11 601.	14 939.6	{8436., 24 819.}	41.7363	0.142767	newly	3992.	4888.41	{2707., 8217.}	40.4684	0.142947	Reff	0.332083	0.377365	{0.253921, 0.558804}	32.8305	ar	92 334.	93 845.1	{93 699., 93 995.}	1.6366
